# Inhibition of Baicalin on Metabolism of Phenacetin, a Probe of CYP1A2, in Human Liver Microsomes and in Rats

**DOI:** 10.1371/journal.pone.0089752

**Published:** 2014-02-26

**Authors:** Na Gao, Bing Qi, Fang-jun Liu, Yan Fang, Jun Zhou, Lin-jing Jia, Hai-ling Qiao

**Affiliations:** 1 Institute of Clinical Pharmacology, Zhengzhou University, Zhengzhou, People’s Republic of China; 2 The 89th Hospital of Chinese People’s Liberation Army, Weifang, People’s Republic of China; Macau University of Science and Technology, Macau

## Abstract

Baicalin has been used as mainly bioactive constituent of about 100 kinds of traditional Chinese medicines in Chinese pharmacopoeia. The effect of baicalin on cytochrome P450 should be paid more attention because baicalin was used widely. The aim of this study was to investigate whether baicalin could inhibit CYP1A2 in pooled human liver microsomes (HLMs) and in rats in vivo and the gene polymorphisms could affect inter-individual variation in IC_50_ in 28 human livers. Phenacetin was used as probe of CYP1A2. Kinetic parameter of CYP1A2 and IC_50_ of baicalin on CYP1A2 to each sample were measured and the common CYP1A2 polymorphisms (−3860G>A and −163C>A) were genotyped. The results showed that baicalin exhibited a mixed-type inhibition in pooled HLMs, with a K_i_ value of 25.4 µM. There was substantial variation in K_m_, V_max_, CL_int_ of CYP1A2 and IC_50_ of baicalin on CYP1A2 (3∼10-fold). The range was from 26.6 to 114.8 µM for K_m_, from 333 to 1330 pmol·min^−1^·mg^−1^protein for V_max_ and from 3.8 to 45.3 µL·min^−1^·mg^−1^ protein for CL_int_ in HLMs (n = 28). The Mean (range) value of IC_50_ in 28 HLMs was 36.3 (18.9 to 56.1) µM. The genotypes of −3860G>A and −163C>A had no significant effect on the inhibition of baicalin on CYP1A2. The animal experiment results showed that baicalin (450 mg/kg, i.v.) significantly decreased the C_max_ and CL of phenacetin, and increased C_60 min_, t_1/2_, V_d_ and AUC (*P*<0.05). There were significant correlations between percentage of control in C_60 min_, t_1/2_, CL, AUC of phenacetin and C_max_ of baicalin in 11 rats (*P*<0.05). Protein binding experiments in vitro showed that baicalin (0–2000 mg/L) increased the unbound phenacetin from 14.5% to 28.3%. In conclusion, baicalin can inhibit the activity of CYP1A2 in HLMs and exhibit large inter-individual variation that has no relationship with gene polymorphism. Baicalin can change the pharmacokinetics of phenacetin in rats.

## Introduction

The consumption of herbal products is becoming increasingly popular as alternative therapies to Western medicine [1]. It was reported that about 80% of the world’s population use herbs to cover their need for drugs [2]. The reason may be that herbal therapeutic efficacy is mild and broad, and the incidence of adverse reactions is relatively low in comparison with synthetic drugs [3]. However, not only the effectiveness of using herbal medicine in combination with modern pharmaceuticals, but also the possible adverse effects from herb–drug interactions remain to be verified [4]. Therefore, it is important to study the interactions between main components of Chinese herbal medicine and pharmaceutical molecules.

Cytochrome P450 (CYP) is the most important phase I metabolic enzyme in liver and metabolize more than 90% of therapeutic drugs [5]. The activity of CYP could be induced and inhibited and the change of CYP activity may lead to change in pharmacological response and/or drug toxicity. It should be emphasized that the inhibition of CYP had been an important cause of drug interactions recognized by the United States Food and Drug Administration (FDA) and other regulatory agencies [6].

Baicalin (5,6,7-trihydroxyflavone-7-β-D-glucuronide) is a main active constituent of Scutellariae Radix, the root of *Scutellaria baicalensis Georgi*, which is widely used in combination with other herbs in Chinese traditional medicines [7], [8]. Moreover, it has been used as a phytochemical marker and mainly bioactive constituent for quality control of about 100 kinds of traditional Chinese medicines in Chinese pharmacopoeia [9]. Our previous studies [9]–[12] had demonstrated that baicalin had different potencies and mechanisms of inhibition on different CYP subtypes. It exhibited the values of K_i_ were 105.6, 155.6, 145.8 and 88.1 µM for CYP3A, CYP2D, CYP2E1 and CYP1A2 in rats, respectively, and inhibition manners were competitive for CYP3A and CYP2E1, non-competitive for CYP2D and mixed-type for CYP1A2, respectively. Moreover the changes in the pharmacokinetics of different probes of different subtypes induced by inhibition of baicalin were different. Baicalin could increase the C_max_ and AUC of dextromethorphan, but it could result in a significant decrease in C_max_ and had no significant effect on AUC of chlorzoxazone. A lot of work about baicalin on CYP in rats was conduct, but the action on human was still unclear so far.

CYP1A2 is an important CYP enzyme subfamily in human beings, accounting for approximately 13% of the total content of this enzyme superfamily [13]. CYP1A2 plays an important role in the metabolism of not only some clinically used drugs including theophylline, clozapine, and tacrine, but also foodborne procarcinogens such as polycyclic aromatic hydrocarbons or imidazoquinoline derivatives [14]. Phenacetin is the preferred probe for screening CYP1A2-based drug interaction potential in vitro [15]. In addition it can be used as an in vivo probe to measure CYP1A2 activity [16], [17]. The human CYP1A2 gene is highly polymorphic. So far, more than 40 SNPs are presented on the CYP allele nomenclature website (http://www.cypalleles.ki.se/cyp1a2.htm). Among them, two polymorphisms have been related to change on CYP1A2 enzymatic activity or inducibility. A genetic polymorphism −3860G>A in the 5′-flanking region of human CYP1A2 gene caused a significant decrease of CYP1A2 activity in Japanese smokers [18]. Another genetic polymorphism in the intron I (−163C>A) associated with higher inducibility [19]. However, it was still unknown whether the CYP1A2 alleles could have similar effects on CYP1A2-related drug inhibition.

The aim of this study was to obtain a clear understanding of the molecular mechanisms underlying inter-individual variation in baicalin inhibition on CYP1A2 through a phenotype–genotype analysis of well-characterized human livers. Phenotype measures included Michaelis constant (K_m_) and maximum velocity (V_max_) and IC_50_. Genotype measures included the known common CYP1A2 polymorphisms (−3860G>A and−163C>A). Moreover, the effects of baicalin on phenacetin pharmacokinetics in rats in vivo and the relationships between pharmacokinetic changes of phenacetin and baicalin concentrations were studied.

## Materials and Methods

### Ethics Statement

The human test was carried out under approval from the Ethics Committee, University of Zhengzhou and all volunteers gave their written informed consent.

This animal test was carried out strictly accordance with the Guide for the Care and Use of Laboratory Animals. All the experimental procedures reported here were reviewed and approved by the Zhengzhou University Animal Care and Use Committee.

### Human Liver Microsomes and Animals

Liver samples from 28 patients with hepatic hemangioma had been obtained from two sources, including the first affiliated hospital and People’s Hospital of Zhengzhou University. All donors with normal liver function were free of infectious diseases. The age range was 20–67 years with 18 samples from female donors and 10 from males. Among them (Table 1), 4 cases had smoking history (defined as 11 cigarettes or more smoking per day) and 4 cases had drinking history (defined as 2∼3 times or more drinking per week). All patients only used routine anesthetics and had no a history of exposure to known CYP- inducing agents and inhibiting agent. Human liver microsomes (HLMs) were prepared by differential centrifugation as previously described [20]. Protein concentrations were measured using Bradfrod method [21].

**Table 1:** Effect of donor gender, age, smoking and drinking on IC_50_ of baicalin for CYP1A2 in HLMs mean±SD.

	Group	n	IC_50_ (µM)
Gender	Male	10	38.3±12.3
	Female	18	35.2±9.5
Age	< = 45	11	36.5±11.6
	>45	17	36.2±10.0
Smoking	Smoking	4	39.8±7.7
	Non-smoking	24	35.7±10.9
Drinking	Drinking	4	39.8±7.7
	Non- drinking	24	35.7±10.9

Male Sprague Dawley (SD) rats weighing 180–220 g were purchased from the Laboratory Animal Center of Henan province. The animals were housed under controlled environmental conditions (lights on from 6∶00 am to 6∶00 pm, temperature 21–22°C, relative humidity 50–60%) and allowed access to a commercial rat chow and tap water *ad libitum*. The animals were allowed to adapt to the environment for at least a week. The rats were fasted 12 h prior to the pharmacokinetic experiments.

### Chemicals and Reagents

Phenacetin and its metabolite, acetaminophen, were obtained from the National Institute for the Food and Drug Control (Beijing, China). Baicalin (>98.5% purity) was donated by Henan Provincial Institute of Food and Drug Control. Methanol and acetonitrile were HPLC grade and purchased from Siyou Chemical Reagent Co. (Tianjin, China). NADPH, reduced nicotinamide adenine dinucleotide phosphate was supplied by Solarbio Science and Technology co. Ltd (Beijing, China). Ultrafiltration tubes (0.5 ml, 10 KD) were purchased from Millipore (USA). Other reagents were of analytical grade.

### The Inhibition of Baicalin on CYP1A2 in HLMs

Kinetic parameter of CYP1A2 and IC_50_ of baicalin to CYP1A2 in HLM from individuals were determined. Moreover the K_i_ value in pooled HLMs was estimate. The CYP1A2 activity was assessed by formation of acetaminophen from phenacetin, a probe substrate. The incubation mixture contained HLMs (0.3 mg/ml), 100 mM phosphate buffer (pH7.4), phenacetin and baicalin at different concentrations with NADPH (1 mM). The mixture was pre-incubated for 5 min at 37°C and the optimal incubation time was 30 min.

For the biotransformations, eight substrate concentrations were examined over the following ranges: 6.25 to 800 µM for phenacetin. K_m_ and V_max_ values of each HLM were determined by nonlinear regression analysis. To estimate the K_i_ value, different concentrations of phenacetin (12.5, 25, 50,100, 200 µM) and baicalin (0, 10, 20, 40, 80 µM) were used in pooled HLMs (n = 9). The 9 individual HLMs were selected according to CYP1A2 genotype and the value of K_m_ from the 28 individual HLMs. The mechanism of inhibition was estimated graphically from Lineweaver–Burk plots. K_i_ value was calculated via second plot of the slopes from Lineweaver–Burk plots versus inhibitor concentrations. Moreover, the substrate concentration was chosen close to K_m_ and IC_50_ of baicalin to CYP1A2 in each HLM was determined.

Termination of the enzyme reaction was by addition of ice-cold acetonitrile. The method of determining acetaminophen, the metabolite of phenacetin, was as follows. The incubation tubes were vortexed and centrifuged then 80 µl clear supernatant was injected to the HPLC system. The mobile phase consisted of methanol and 0.05 M ammonium acetate (20∶80, v/v) at a flow rate of 1 ml·min^−1^. The UV detection wavelength was 257 nm.

### Genotyping the −163C>A and −3860G>A Polymorphisms in CYP1A2 Gene

Genomic DNA was isolated from human liver tissue. Genotyping of the −163C>A and −3860G>A polymorphisms were performed by the polymerase chain reaction (PCR)-restriction fragment length polymorphism (RFLP) methods described previously or PCR-sequencing [18], [22]. The sequences of upstream and downstream primer of −163C>A polymorphism were 5′-GGA AGG TAT CAG CAG AAA GCC-3′ and 5′-GGC TCA TCC TTG ACA GTG CC-3′, respectively. The sequences of upstream and downstream primer of −3860G>A polymorphism were 5′-GCT ACA CAT GAT CGA GCT ATA C-3′ and 5′-CAG GTC TCT TCA CTG TAA AGT TA-3′, respectively.

### Effects of Baicalin on Phenacetin Pharmacokinetics in Rats in vivo

Sprague–Dawley rats were chosen to conduct this experiment and drug dosing was done via the tail vein. The study was based on a randomized, two-period crossover design at intervals of 4 days. Eleven rats were randomly divided into two groups. Group 1 included 6 rats and group 2 included 5 rats. During the phase I, the rats in group 1 were treated with normal saline (control) and the rats in group 2 were treated with baicalin (450 mg/kg, i.v.). After that an i.v. dose (5 mg/kg) of phenacetin was given immediately. Blood samples were collected before and at 0, 5, 15, 30, 60, 90 and 120 min after administration by orbital bleeding via heparinized capillary tubes. The sample at 0 min was collected immediately after i.v. injection of phenacetin. Plasma was separated from the blood by centrifugation at 4000 rpm for 10 min and was stored at −30°C until analyzed. After a washout period of 4 days, the two groups crossed over to receive the alternative drug.

### Determination of Plasma Phenacetin and Baicalin Concentration

Plasma concentration of phenacetin was determined by HPLC-UV. 1 ml acetic ether was added to 0.1 ml of plasma from each sample and vortexed for 2 min. The samples were centrifuged and the organic phase was evaporated to dryness under a stream of nitrogen. The residue was reconstituted in 100 µl of mobile phase and 50 µl was injected to the HPLC system. The mobile phase consisted of methanol and water (51∶49, v/v) at a flow rate of 1 ml·min^−1^. The UV detection wavelength was 247 nm.

The method of determining plasma baicalin concentration had been reported previously [11].

### Measurement of Rat Plasma Protein Binding of Phenacetin in vitro

The effect of baicalin on protein binding of phenacetin in fresh rat plasma (*n* = 5) was measured in vitro.

The final phenacetin concentration was 7 mg/L and baicalin concentrations varied from 0 to 2000 mg·L^−1^ in plasma samples. The samples were incubated for 30 min at 37°C and were placed into an ultrafiltration tubes. The samples were centrifuged at 4500 rpm for 15 min. Concentration of phenacetin in the filtrate was determined by the method described above.

### Statistical Analysis

Pharmacokinetic parameters were calculated by DAS 2.0 (Mathematical Pharmacology Professional Committee of China, Shanghai, China). Statistical analysis was performed using SPSS 17.0 software (SPSS Inc., Chicago, IL, USA). IC_50_ of HLMs between different groups was compared using independent samples *t*-test or analysis of variance. Correlation coefficients and statistical significance were determined using Pearson test. The influences of baicalin on changes in pharmacokinetics parameters of phenacetin were evaluated by Paired samples *t* test. A *P* value less than 0.05 was considered statistically significant.

## Results

### Inhibition of Baicalin on CYP1A2 in HLMs

#### The Ki of baicalin on CYP1A2 in pooled HLMs

**Figure 1:**
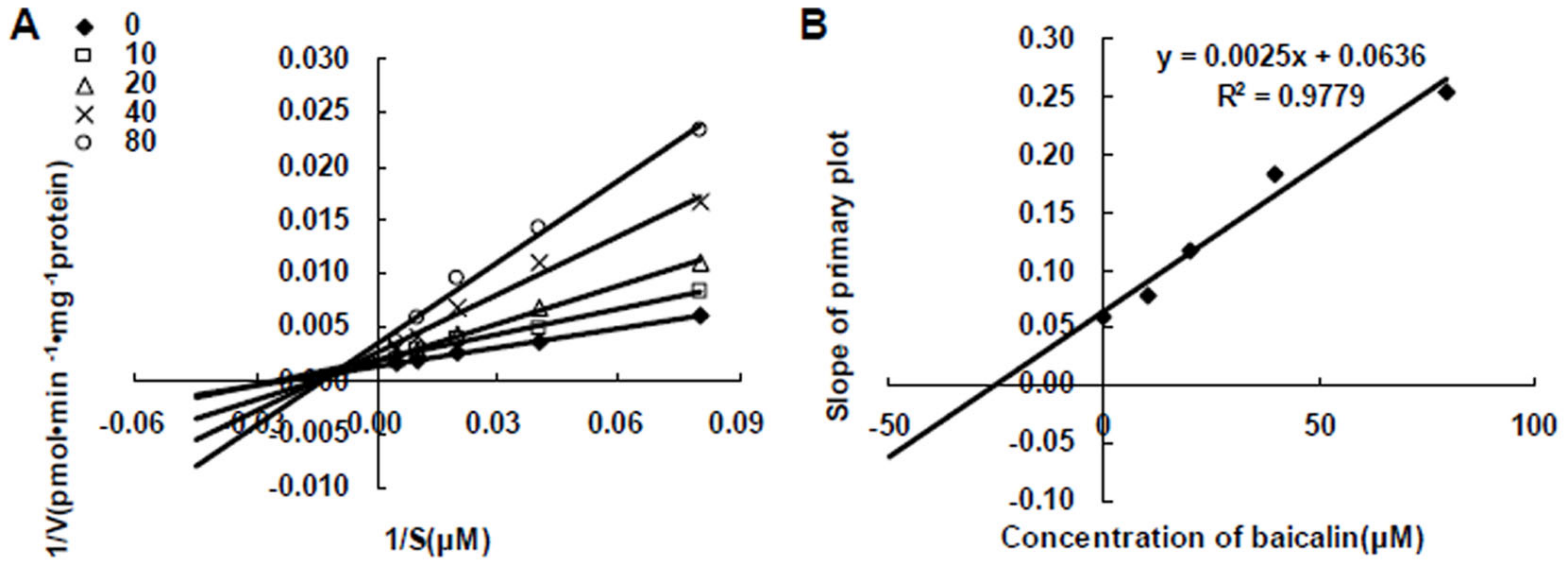
Inhibition of CYP1A2 activity by baicalin in pooled human liver microsomes. (A) Lineweaver-Burk plots of the effect of baicalin on formation of acetaminophen in pooled human liver microsomes. Reactions were performed in the presence of phenacetin (12.5, 25, 50, 100, 200 µM) and various concentrations of baicalin (0, 10, 20, 40, 80 µM) in the microsome (0.3 mg/mL). (B) Secondary plot of the slopes from the Lineweaver–Burk plots verse baicalin concentrations.

To characterize the kinetics of CYP1A2 enzyme inhibition by baicalin, the assay was conducted with multiple concentrations of baicalin and multiple concentrations of the substrates. Lineweaver–Burk plots for the inhibition of CYP1A2 were shown in Figure 1A. Based on nonlinear regression analysis of the enzyme kinetic data, the mode of inhibition of baicalin on CYP1A2 was mixed type inhibited with a K_i_ value of 25.4 µM (Figure 1B).

#### The IC50 of baicalin on CYP1A2 in 28 HLMs

K_m_ and V_max_ values of CYP1A2 in each HLM were shown in Table 2. The range were from 26.6 to 114.8 µM for K_m_, from 333 to 1330 pmol·min^−1^·mg^−1^protein for V_max_ and from 3.8 to 45.3 µL·min^−1^·mg^−1^ protein for CL_int_. In order to study the inhibition of baicalin on CYP1A2 in each HLM, substrate concentration was chosen approximately to K_m_. The Mean (range) value of IC_50_ in 28 HLMs was 36.3 (18.9 to 56.1) µM. Inter-individual variation was about 3-fold.

**Table 2:** The K_m_, V_max_ and CL_int_ for CYP1A2 and IC_50_ of baicalin on CYP1A2 in HLMs (n = 28).

	K_m_(µM)	V_max_(pmol·min^−1^·mg^−1^pro)	CL_int_(µL·min^−1^·mg^−1^ pro)	IC_50_(µM)
Range	26.6∼114.8	333∼1330	3.8∼45.3	18.9∼56.1
Mean±SD	52.3±21.5	830.8±270.8	18.3±9.1	36.3±10.5
95%CI	10.2∼94.4	300∼1361.6	0.5∼36.1	15.7∼56.9

#### Effects of donor gender, age, smoking and drinking on the inhibition of baicalin in HLMs

The effects of donor gender, age, smoking and drinking on the inhibition of baicalin in HLMs were showed in Table 1. The results showed that there were no significant differences between different groups.

#### Effects of gene polymorphisms on inhibition of baicalin in HLMs

We assessed the relationships between −3860G>A, −163C>A alleles and haplotypes, and the values of IC_50_ (Table 3). The results showed that there were no significant effects of the polymorphisms on IC_50_ of baicalin.

**Table 3:** Effects of SNP −3860G>A and −163C>A on IC_50_ of baicalin for CYP1A2 in HLMs mean±SD.

	Genotype	n	IC_50_ (µM)
−3860G>A	G/G	15	37.2±10.4
	G/A	8	33.3±9.4
	A/A	5	38.6±13.4
−163C>A	C/C	4	38.1±10.1
	C/A	10	36.3±12.9
	A/A	14	35.8±9.4
haplotypes	−3860G/G−163C/C	4	38.1±10.1
	−3860G/G−163C/A	5	38.9±14.5
	−3860G/G−163A/A	6	35.1±8.2
	−3860G/A−163C/A	5	33.7±12.1
	−3860G/A−163A/A	3	32.8±3.7
	−3860A/A−163A/A	5	38.6±13.4
Total		28	36.3±10.5

### Effect of Baicalin on CYP1A2 in Rats in vivo

#### Pharmacokintics of baicalin in rats

**Figure 2:**
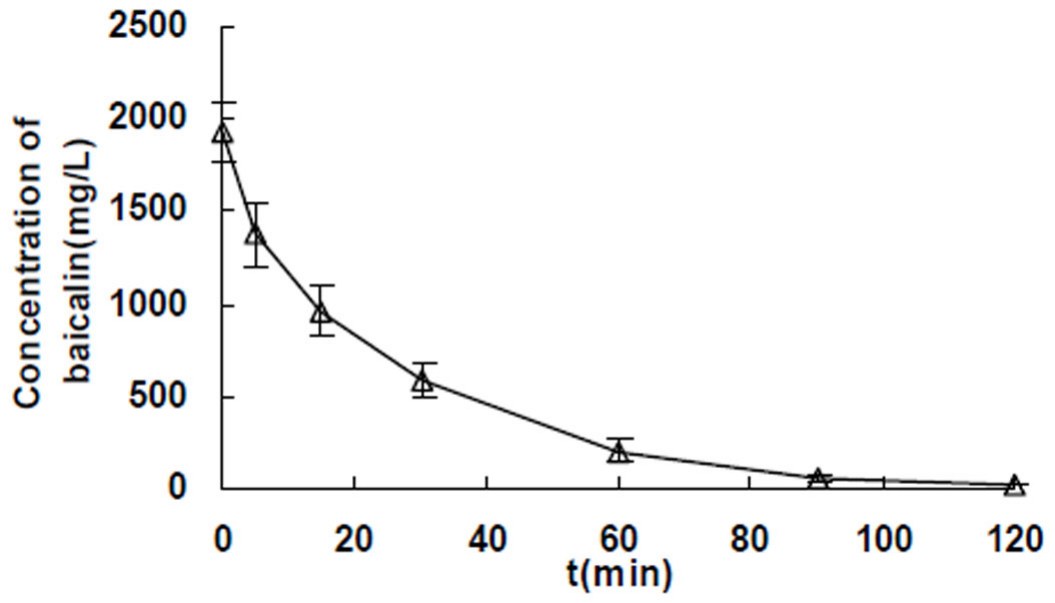
Mean plasma concentration–time profile of baicalin combination with phenacetin after i.v. administration baicalin at 450 mg/kg in rats. Each point represents the mean±SD (n = 11).

The baicalin plasma concentration–time curve was shown in Figure 2. The C_max_ and AUC in rats treated with baicalin (450 mg/kg) were (1934±164) mg/L and (48.7±6.1) g/L**·**min, respectively.

#### Effects of baicalin treatment on phenacetin pharmacokinetics

**Figure 3:**
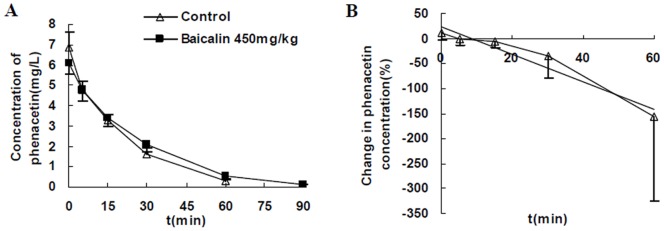
Effects of baicalin treatment on phenacetin pharmacokinetics. (A) The concentration-time profiles of phenacetin (5 mg/kg,i.v.) after treatment with normal saline (control) or baicalin (450 mg/kg, i.v.) in rats. (B) The changes in concentrations(%)-time profile of phenacetin (5 mg/kg, i.v.) after treatment with baicalin (450 mg/kg, i.v.) compared with control. Each point represents the mean±SD (n = 11).

Pharmacokinetics of phenacetin: The plasma concentration versus time profile of phenacetin obtained in the pharmacokinetic studies was given in Figure 3A. This clearly illustrated that the concentration of phenacetin was too low to be detectable at 90 min after administration in control, while it was still (0.12±0.02) mg·L^−1^ in rats treated with baicalin. As shown in Table 4, baicalin (450 mg/kg, i.v.) was found to significantly decrease the C_max_ and CL of phenacetin, and increase C_60 min_, t_1/2_, V_d_ and AUC (*P*<0.05). The AUC and C_60 min_ of phenacetin in control were (140.2±14.7) mg/L**·**min and (0.27±0.14) mg/L compared with (162.8±21.1) mg/L**·**min and (0.55±0.17) mg/L in rats treated with baicalin (450 mg/kg), respectively. Co-administration of baicalin increased the mean AUC of phenacetin by 16%, and the mean C_60 min_ of phenacetin by 104%.

**Table 4:** Pharmacokinetic parameters of phenacetin (5 mg/kg, i.v.) after treatment with baicalin (450 mg/kg, i.v.).

	control	Baicalin
C_max_(mg/L)	6.90±0.72	6.10±0.58*
C_60 min_(mg/L)	0.27±0.14	0.55±0.17**
t_1/2_(min)	14.0±2.3	18.5±2.7**
V_d_(L/kg)	0.72±0.07	0.81±0.07*
CL(L/min/kg)	0.036±0.004	0.031±0.004*
AUC(mg/L**·**min)	140.2±14.7	162.8±21.1*

*P<0.05 vs control,

**P<0.01 vs control.

The changes in phenacetin concentrations (%) at each sampling time of each rat after treatment with baicalin could be calculated because of crossover design. The changes (%) versus time profile was shown in Figure 3B, which showed a strong correlation (r = −0.97, *P*<0.01). It clearly illustrated that phenacetin concentration decreased slightly at first and increased significantly subsequently after treatment with baicalin.

**Figure 4:**
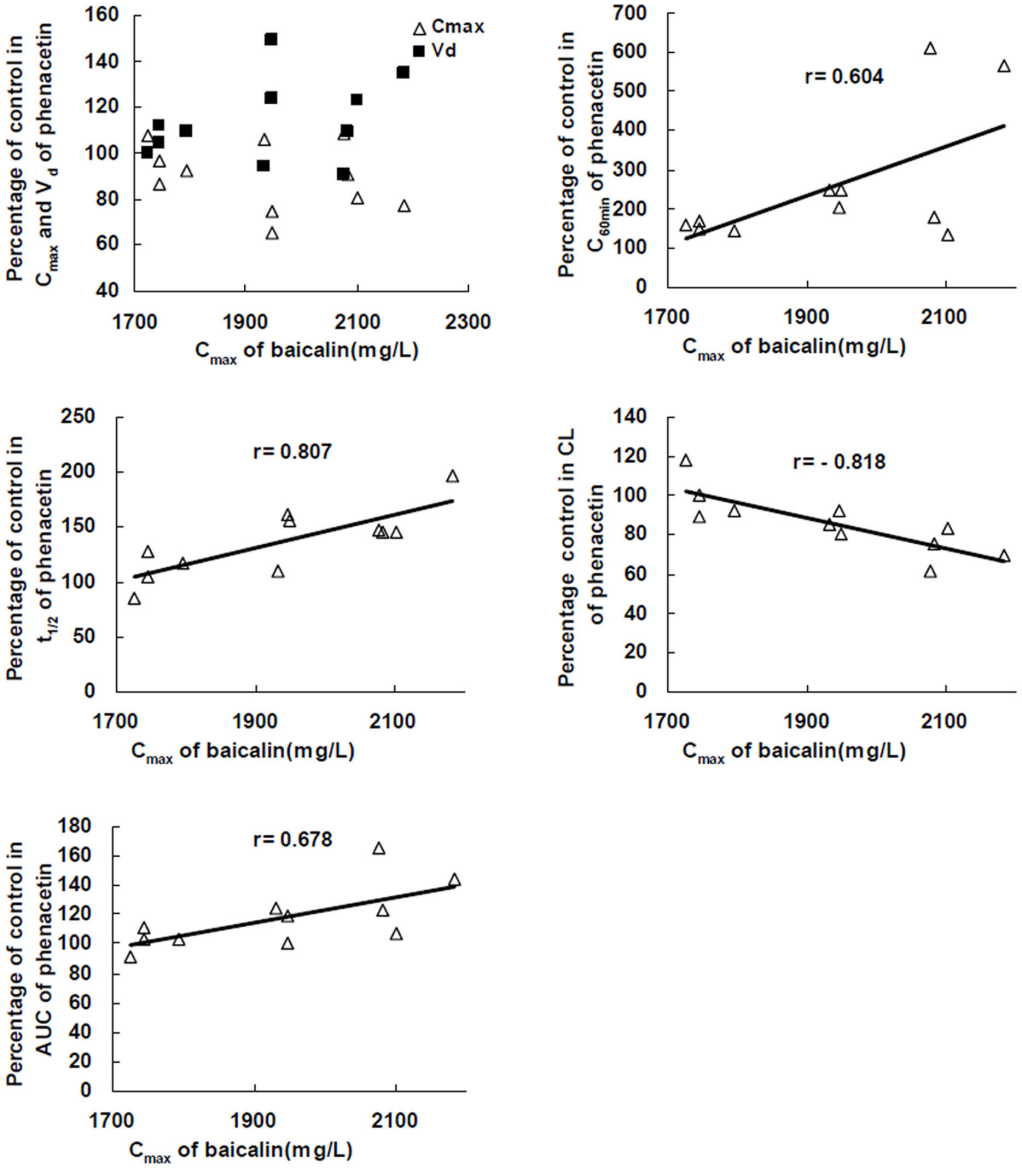
Plots of percentage of control in pharmacokinetic parameters of phenacetin versus C_max_ of baicalin.

**Figure 5:**
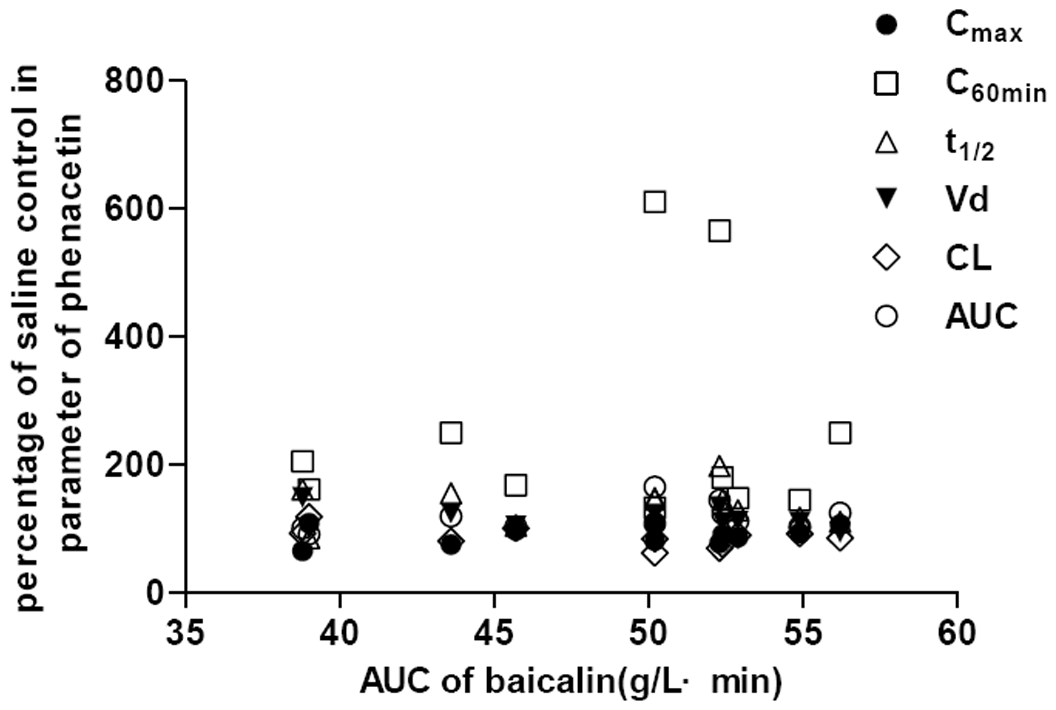
Plots of percentage of control in pharmacokinetic parameters of phenacetin versus AUC of baicalin.

Correlations of the changes in phenacetin pharmacokinetic parameters and baicalin: The study was a randomized, two-period crossover design, so the changes in pharmacokinetic parameters of each rat could be calculated. There were significantly differences in C_max_, C_60 min_, t_1/2_, V_d_, CL and AUC of phenacetin between rats treated with normal saline and baicalin, so the correlations of this change (%) in parameters and C_max_ or AUC of baicalin could be analyzed. The results showed that except C_max_ and V_d_, there were significant correlations between percentage of control in other pharmacokinetic parameters of phenacetin and C_max_ of baicalin in 11 rats (*P*<0.05, Figure 4). In addition, there were no significant correlations between percentage of control in all pharmacokinetic parameters of phenacetin and AUC of baicalin (Figure 5).

#### Effect of baicalin on rat plasma protein binding of phenacetin in vitro

**Figure 6:**
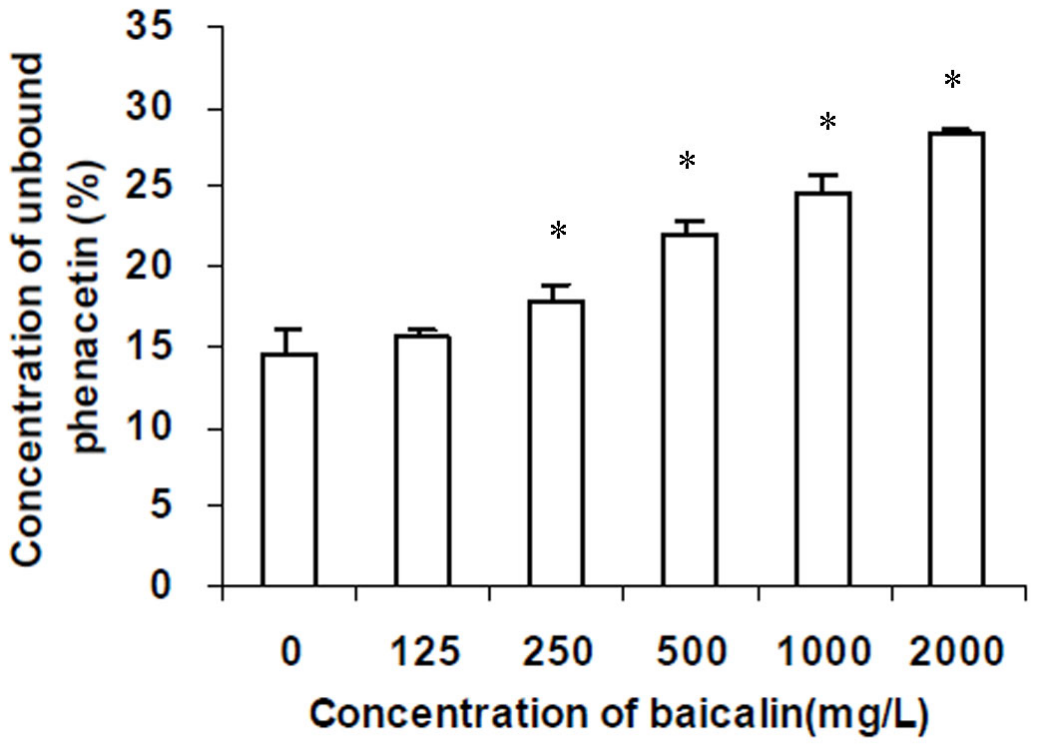
Effect of baicalin at concentration ranged 0 from to 2000·L^−1^ on concentration of unbound phenacetin(%) in pooled rat plasma (n = 3). Total phenacetin concentration was 7·L^−1^. *indicates a significant (*P*<0.05) increase in concentration of unbound phenacetin (%) from blank value.

In order to explain why C_max_ of phenacetin decreased after treatment with baicalin, the effect of baicalin on rat plasma protein binding of phenacetin in vitro was studied. As shown in Figure 6, the unbound phenacetin (%) was 14.5%. It increased significantly when the concentration of baicalin was 250 mg·L^−1^. When the concentration of baicalin was 2000 mg·L^−1^, which was equivalent to the C_max_ value in rat treated with baicalin (450 mg/kg), the concentration of unbound phenacetin (%) increased approximately 2-fold.

## Discussion

In this study, we focused the inhibition of baicalin on CYP1A2 in HLMs by using phenacetin, a probe of CYP1A2. The results showed that the mode of inhibition of baicalin on CYP1A2 was mixed type inhibited with a K_i_ value of 25.4 µM. The result indicated that the inhibition on CYP1A2 in HLMs was much stronger than that in RLMs (K_i_ = 88.1 µM). Moreover we studied the effect of baicalin on the pharmacokinetics of phenacetin in rats because phenacetin was used in China only as a component of compound. The results demonstrated that the AUC was significant increased and CL was significant decreased after treatment with baicalin (450 mg/kg). As a much stronger inhibitor of CYP1A2 in HLM, it should be paid more attention to the pharmacokinetic changes of phenacetin induced by baicalin in clinic. Our previous results showed that the AUC of theophylline had no significant change after treated by baicalin (450 mg/kg was administered at 0 h), a probe of CYP1A2 [11]. The results indicated that the inhibition of baicalin on the substrate of CYP1A2 was specific and the inhibition potency was stronger on phenacetin than that on theophylline.

Our results showed that the value of K_i_ of baicalin on CYP1A2 in HLMs was 25.4 µM. Inhibition data in vitro may be used to rank order the inhibition of particular CYPs in order to test the clinical relevance for the most likely affected CYP. It was estimated that interactions are possible if the ratio of inhibitor C_max_/K_i_ is between 1 and 0.1 [15]. Ju et al [23] had reported that after i.v. infusion of Yinhuang injection which contains 84 mg baicalin to the healthy volunteers, the C_max_ of baicalin was about 3.36 µM. It was reported that there was proportionality between dosage and C_max_ of baicalin [10]. Furthermore the daily dose of baicalin in Yinzhihuang injection is 450 mg and the estimated C_max_ of baicalin was about 18 µM. The ratio of inhibitor C_max_/K_i_ was about 0.71. So it was necessary to conduct the study that the effect of baicalin on CYP1A2 activity in vivo. Our results in vivo showed that baicalin significantly inhibited the metabolism of phenacetin (*P*<0.05). Since phenacetin is a probe of CYP1A2, the clinical interaction between baicalin and other CYP1A2 substrates should be paid more attention. The results in vivo showed that the AUC only increased by 16% and CL only decrease by 14% in rats after treatment with baicalin (*P*<0.05). It meant that the inhibition of baicalin in vivo seemed weak. This phenomenon might be related with displacement of phenacetin from plasma protein by baicalin [10], [11].

Correlation analysis showed that there were significant correlations between percentage of control in C_60 min_, t_1/2_, CL, AUC of phenacetin and C_max_ instead of AUC of baicalin in 11 rats. The results demonstrated that the changes in pharmacokinetic parameters of phenacetin may be related with delivery speed. Moreover, the changes in phenacetin concentrations (%) at different sampling time after treatment with baicalin had significant correlation with sampling time, which mean that the increase of phenacetin concentration was more and more obvious with the time prolonging.

Gene polymorphisms in CYP enzymes may affect not only CYP-mediated drug metabolism but also drug inhibition. Genotype-dependent drug inhibition of CYP2C19 and CYP2C9 had been demonstrated in clinical studies. Overall, EMs (Extensive metabolizers) of CYP2C19 experienced a greater degree of drug-drug interactions (DDIs) compared with PMs (Poor metabolizers) [24], [25]. CYP2C9*3 was less potently inhibited than wild-type CYP2C9 in both *in vitro* and *in vivo* DDI studies [26], [27]. To evaluate the impact of CYP1A2 polymorphic enzymes on in vitro drug inhibitory potential, the IC_50_ values of baicalin in each HLM were determined. The results showed that there was significant inter-individual variation in the IC_50_ values (3.0-folds). The most extensively studied polymorphisms are −3860G>A (CYP1A2*1C), −2467delT (CYP1A2*1D), −739T>G (CYP1A2*1E) and −163C>A [28]. Among them the incidence of allele A at −3860 in the 5′-flanking region and allele C at −163 in the intron I were 22∼25% and 32∼34% in Chinese, respectively [29]. Our results showed that the two polymorphisms had no effect on inhibition of baicalin. Moreover, our results also showed that there were no effects of gender, age, smoking and alcohol on this inhibition. The variation in IC_50_ of baicalin on CYP1A2 in HLMs may also be due to other as yet unknown mutations or due to environmental factors. Further studies were required to identify the mechanisms behind this phenomenon. It was reported that CYP1A2 mRNA content showed an up to 40-fold variation and protein level varied 3 to 30 folds between individuals [30], [31]. Whether the variation in the IC_50_ was related with the content of CYP1A2 should be studied in the future.

In conclusions, in this study we demonstrated that baicalin can inhibit the activity of CYP1A2 in HLMs and exhibit large inter-individual variation that has no relationship with the gene polymorphisms. Baicalin can change the pharmacokinetics of phenacetin in rats.
